# Public knowledge, attitudes, practices, and barriers to skin cancer screening in the United Arab Emirates

**DOI:** 10.1371/journal.pone.0316613

**Published:** 2025-01-31

**Authors:** Anan S. Jarab, Walid Al-Qerem, Karem H. Alzoubi, Mariam Al Mohammad, Shrouq R. Abu Heshmeh, Yazid N. Al Hamarneh, Tareq Mukattash, Maher Khdour

**Affiliations:** 1 College of Pharmacy, Al Ain University, Abu Dhabi, United Arab Emirates; 2 Faculty of Pharmacy, Department of Clinical Pharmacy, Jordan University of Science and Technology, Irbid, Jordan; 3 Faculty of Pharmacy, Department of Pharmacy, Al-Zaytoonah University of Jordan, Amman, Jordan; 4 Department of Pharmacy Practice and Pharmacotherapeutics, University of Sharjah, Sharjah, United Arab Emirates; 5 Faculty of Pharmacy, Jordan University of Science and Technology, Irbid, Jordan; 6 Faculty of Medicine and Dentistry, Department of Pharmacology, University of Alberta, Edmonton, Canada; 7 College of Pharmacy, Al-Quds University, Abu Dis, Palestine; Ministry of Health, General Health Directorate of Raparin and University of Raparin, IRAQ

## Abstract

**Objective:**

To assess knowledge, attitude, practice, and barriers to skin cancer screening among the general population of the United Arab Emirates (UAE).

**Methods:**

In this cross-sectional study, an online-based questionnaire was distributed via online social media sites such as Facebook, WhatsApp, Twitter, and others to the residents of the UAE. The questionnaire evaluated socio-demographics, knowledge (8 items), attitudes (10 items), practices (14 items), and barriers (9 items) in the context of skin cancer screening. A binary logistic regression was conducted to assess variables associated with practice levels.

**Results:**

The study included 924 participants (51.9% females), with a median (IQR) age of 32 (22–44) years. Results showed a window for knowledge improvement [median = 4 (3–6) out of a maximum potential score of 8], unfavourable attitudes [median = 37 (33–39) out of a maximum potential score of 50], inadequate practices [median = 41 (31–45) out of a maximum possible score of 70]. The most common barriers to skin cancer screening were lack of knowledge about skin cancer (74.1%), lack of awareness of the need for skin cancer screening (72.2%), and absence of symptoms (54.1%). Older age (OR = 0.985, 95%CI: 0.971–0.998) and lack of health insurance (OR = 0.478, 95%CI: 0.301–0.758) were associated with lower screening practices. Female gender (OR = 1.833, 95%CI: 1.361–2.469), being married (OR = 1.415, 95%CI: 1.006–1.992), being a non-smoker (OR = 1.568, 95%CI: 1.045–2.352) or a former smoker (OR = 2.555, 95% CI: 1.082–6.034), and more favourable attitudes (OR = 1.071, 95%CI: 1.044–1.096) were associated with higher screening practices.

**Conclusion:**

The UAE population exhibited moderate knowledge, unfavourable attitudes, and inadequate practices regarding skin cancer screening, with several barriers identified. Targeted online and in-person awareness campaigns are crucial for improving public understanding and attitudes, thereby enhancing screening practices, especially among older adults, males, smokers, and those without health insurance.

## Introduction

Skin cancer is an increasing public health issue, consisting of two major types: non-melanoma skin cancer, which includes squamous cell carcinoma and basal cell carcinoma, and malignant melanoma. Squamous cell carcinoma and basal cell carcinoma are the most prevalent forms, while malignant melanoma, though less common, is the leading cause of skin cancer-related deaths [1]. Signs and symptoms of skin cancer include the appearance of new moles or growths on the skin, change in size, shape or color of existing moles, spots or moles that contain multiple colors, itchy or bleeding moles, and unhealed skin sores. Risk factors include a family history of skin cancer, increased age, a weakened immune system, increased exposure to ultraviolet radiation (UV) radiation from the sun or tanning beds, and having fair skin, light hair and light eyes [1–4]. Skin cancer treatment varies by type and stage and involves surgical excision, cryotherapy, topical chemotherapy, and immunotherapy in advanced cases [2]. Dermatologists recommend moisturizers and hydrating products such as Neutrogena Hydro Boost Water Gel or CeraVe Daily Moisturizing Lotion, and broad-spectrum sunscreen with sun protection factor (SPF) 30 or higher such as Neutrogena Ultra Sheer Dry-Touch Sunscreen to prevent further damage [2].

Skin cancer is the world’s 17th most common cancer, with over 331,722 new cases diagnosed in 2022 [5]. It has been estimated that approximately 5.4 million basal and squamous cell skin cancers are diagnosed each year in the United States [6]. Age-specific and overall age-standardized incidence rates of melanoma in North Africa and the Middle East region were estimated at 1.66 per 100,000 person-years [7,8]. In the United Arab Emirates (UAE), skin cancer is the fourth most common type of cancer, following breast, thyroid, and colorectal cancers [9]. In 2019, skin cancer accounted for 6.3% of all malignant cases in the UAE population [9].

Given the projected increase in the burden of skin cancer, early detection and screening are vital tools in the battle against skin cancer, both globally and regionally [10]. Despite the availability of cancer screening tools, data from UAE Ministry of Health and Prevention indicate poor screening practices for different types of cancer [11]. This low uptake may be attributed to insufficient public awareness, challenges in accessing cancer screening centres, and various psychosocial, cultural, socioeconomic, healthcare system, or healthcare provider-related factors [12]. In the UAE, cultural norms might discourage women from seeking preventive care or discussing health issues openly. The tradition full-body coverage does not allow for inspecting changes in the skin regularly, probably influencing awareness about skin health. Additionally, the cultural preference for tanned skin through sunbathing without protection might lead to lower awareness of the risks associated with UV exposure, particularly in a region with intense sunlight like the UAE. Furthermore, the UAE population is diverse, including both locals and residents with various skin types, who might underestimate the risk of skin cancer due to misconception that it primarily affects individuals with lighter skin tones.

Earlier studies have demonstrated a high level of general awareness of skin cancer. One study reported that 61.2% of respondents were aware of the causes and effects of skin cancer [13]. A recent systemic review revealed moderate knowledge about skin cancer among 2,841 nursing students who participated in five cross-sectional studies [14]. In Saudi Arabia, one study revealed that the majority of participants correctly identified the symptoms, risk factors, and preventive measures for skin cancer [15]. Furthermore, another study showed that 73% of participants were aware of the association between skin cancer and sunlight exposure but did not recognize the risks associated with moderate tanning [16]. However, while many published studies indicated a reasonable level of knowledge regarding skin cancer, this knowledge has not translated into behaviour [17,18]. Additionally, although previous research has focused on evaluating knowledge and awareness of various aspects of skin cancer, it has not addressed perceptions of the disease and its influence on screening practices and the associated factors, highlighting the need for further exploration [19].

In the UAE, few studies have investigated the barriers to cancer screening, primarily focusing on breast and colorectal cancers. However, there are no studies addressing the barriers to skin cancer screening among the public in the UAE. Therefore, the aim of this study was to assess the knowledge, attitude, practice, and barriers regarding skin cancer screening among the general population of the UAE, and to explore the factors associated with high practice levels. The findings of this study should inform the development of future health initiatives and educational campaigns aimed at increasing public awareness and improving behaviours and practices related to skin cancer screening.

## Methods

### Study design and participants

The data collection in this cross-sectional study was conducted between May 10^th^ and July 10^th^, 2024. The questionnaire was disseminated as a Google Form via various online platforms, including social media websites such as Facebook, WhatsApp, and Instagram, targeting the general population of the UAE using a convenience sampling technique. These reputable online platforms support encryption and security features to protect data during transmission and storage. Additionally, the survey collected anonymous responses and did not collect personal identifiers such as names, email addresses or telephone numbers. Furthermore, the data was securely stored on password-protected servers at the principal investigator’s office. The survey was posted on various online platforms and communities to recruit participants from different backgrounds and to emphasize the diversity of the participants. Additionally, we adjusted our data collection approach by using targeted ads and leveraging community groups relevant to our research topic to better include underrepresented groups based on tracked response rates across different demographics. Participants who were 18 years of age or older were eligible to participate in the study. Exclusion criteria included individuals under 18 years old and those not living in the UAE. It took an average of ten minutes to complete the study questionnaire.

### Study instrument

Following a review of the relevant literature [1–3,20–24], the study questionnaire was developed. An expert panel assessed the questionnaire’s content validity and made the necessary revisions. A panel of experts, including two oncologists and two professors in public health, evaluated the comprehensiveness and relevance of the study questionnaire. A pilot study was conducted with twenty individuals to assess the survey’s completion time, relevance, and clarity. Based on feedback from the pilot study, we revised ambiguous questions to enhance clarity and eliminated questions that participants found repetitive. The pilot test data were not incorporated into the final analysis. The Cronbach’s alpha of the attitude and practice scales was 0.76 and 0.87, respectively demonstrating the reliability of the study instrument. The survey began with a brief introduction outlining the objectives of the study, assuring participants of the voluntary nature of participation and that they could withdraw at any time, even after consenting. The introduction also emphasized the anonymity of participants and the confidentiality of the study findings. The first section of the survey gathered sociodemographic data, along with information on family history of skin cancer or other types of cancer, personal history of skin cancer, any prior skin cancer screenings, major sources of information about skin cancer, and reasons for performing skin self-examinations. The second section evaluated participants’ knowledge of skin cancer screening (8 items), risk factors for skin cancer (11 items), and the signs and symptoms of the disease (12 items). Attitudes toward skin cancer screening were evaluated using a 5-point Likert scale, with 1 point for "strongly disagree" and 5 points for "strongly agree." Participants’ responses regarding skin cancer screening-related practices were also assessed on a 5-point Likert scale, ranging from “never” (1 point) to “always” (5 points). The last part of the questionnaire included nine items that assessed the barriers to skin cancer screening. -All questions in the questionnaire were mandatory, and the Google Form setting required completion of all mandatory questions before submission. Therefore, all submitted forms were complete.

### Sample size calculations

The Krejcie and Morgan formula was used to determine the minimal required sample size: S = X2NP (1—P) + a2(N − 1) + X2P (1- P) [25], where S = required sample size, X2 = the table value of chi-square for 1 degree of freedom at the desired confidence level (3.841), N = the population size, P = the population proportion (assumed to be 50 to provide the maximum sample size), and d = the degree of accuracy expressed as a proportion (0.5). The current study assumed an indefinite population to calculate the required sample size, as the exact number of individuals meeting the inclusion criteria is unknown. Therefore, the minimum required sample size was 384 for a 95% confidence level (CI) and a 5% margin of error. The survey was distributed to a larger number of participants than the calculated sample size to account for potential non-responses and to enhance the confidence and reliability of the study findings.

### Statistical analysis

Data analysis was conducted using the Statistical Package for Social Sciences (SPSS, version 28, Illinois, New York, USA). Categorical variables were presented as frequencies and percentages. Considering the non-normal distribution of continuous data, continuous variables were presented as medians with 25–75 percentiles. Participants’ screening practices were classified as “high” for those who scored above the median practice score and “low” for those who scored below the median. Since the outcome was dichotomous (high vs. low screen practice groups), binary logistic regression was conducted to identify the variables associated with skin cancer screening practices. The independent variables included age, gender, income and marital statuses, educational level, health insurance coverage, smoking status, source of information about skin cancer (scientific vs. non-scientific), and knowledge and attitude scores. Model fit was assessed using the Hosmer and Lemeshow goodness-of-fit test. Statistical significance was set at a threshold of p < 0.05.

### Ethical approval statement

The study received ethical approval from the research ethics committee at Al Ain University- Abu-Dhabi Campus (Ref #: COP/AREC/AD/40). Prior to starting the survey questionnaire, the participant individuals were required to give consent by checking a box indicating they had read the study information and agreed to participate. This served as informed consent to participate.

## Results

The present study included 924 participants, with a median (IQR) age of 32 (22–44) years. Most of the participants were female (51.9%), married (54.9%), completed a bachelor’s degree (62.0%), and did not smoke (80.1%). In total, 37.8% of participants had no income. Most of the participants had health insurance (88.1%) and did not have a family history of skin cancer (99.1%). Furthermore, most of the participants never had skin cancer (97.6%) and did not perform an early skin cancer screening (51.9%). Table 1 displays participants’ sociodemographic and disease-related profiles.

**Table 1:** Socio-demographic characteristics of the study participants (924).

		Frequency (%) or median (25–75) percentiles
Age		32 (22–44)
Gender	Female	480 (51.9%)
	Male	444 (48.1%)
Marital Status	Married	507 (54.9%)
	Single/widowed/divorced	417 (45.1%)
Educational Level	High school or less	143 (15.5%)
	Diploma	110 (11.9%)
	Bachelor	573 (62.0%)
	Postgraduates (Master/PhD)	98 (10.6%)
Monthly income	No income	349 (37.8%)
	< 10,000 AED	182 (19.7%)
	10,000 AED to 20,000 AED	243 (26.3%)
	>20,000 AED	150 (16.2%)
Health insurance coverage	No	110 (11.9%)
	Yes	814 (88.1%)
Smoking	No	40 (80.1%)
	Ex-smoker	31 (3.4%)
	Yes	153 (16.6%)
Family history of skin cancer	No	916 (99.1%)
	Yes	8 (0.9%)
Family history of cancer other than skin cancer	No	844 (91.3%)
	Yes	80 (8.7%)
Have you ever had skin cancer?	No	902 (97.6%)
	Yes	22 (2.4%)
Have you performed a screening for skin cancer before?	No	480 (51.9%)
	Yes	444 (48.1%)

**Fig 1:**
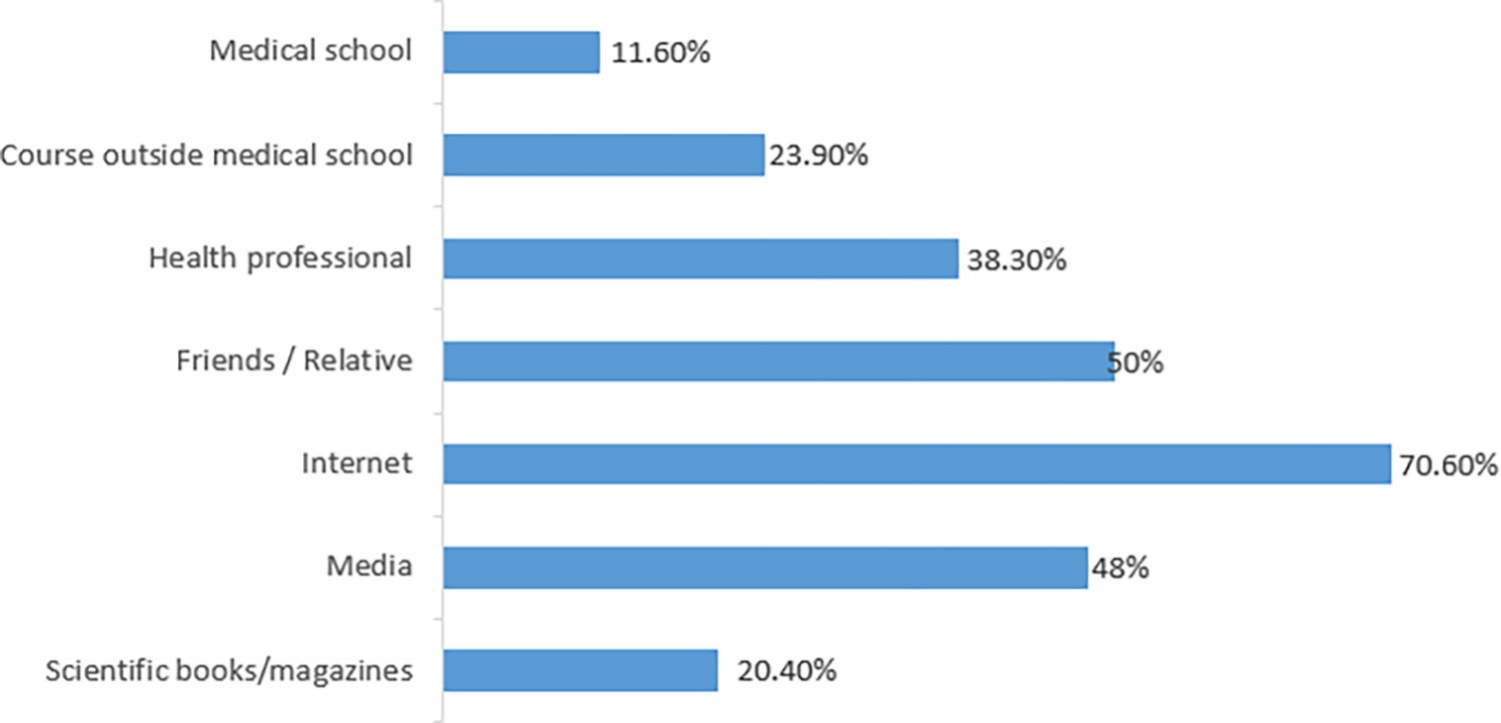
Major sources of information about skin cancer.

As shown in Fig 1, the most frequently reported information source was the internet (69.0%), followed by friends or relatives (49.9%), while medical school represented the least commonly reported source (12.1%).

**Fig 2:**
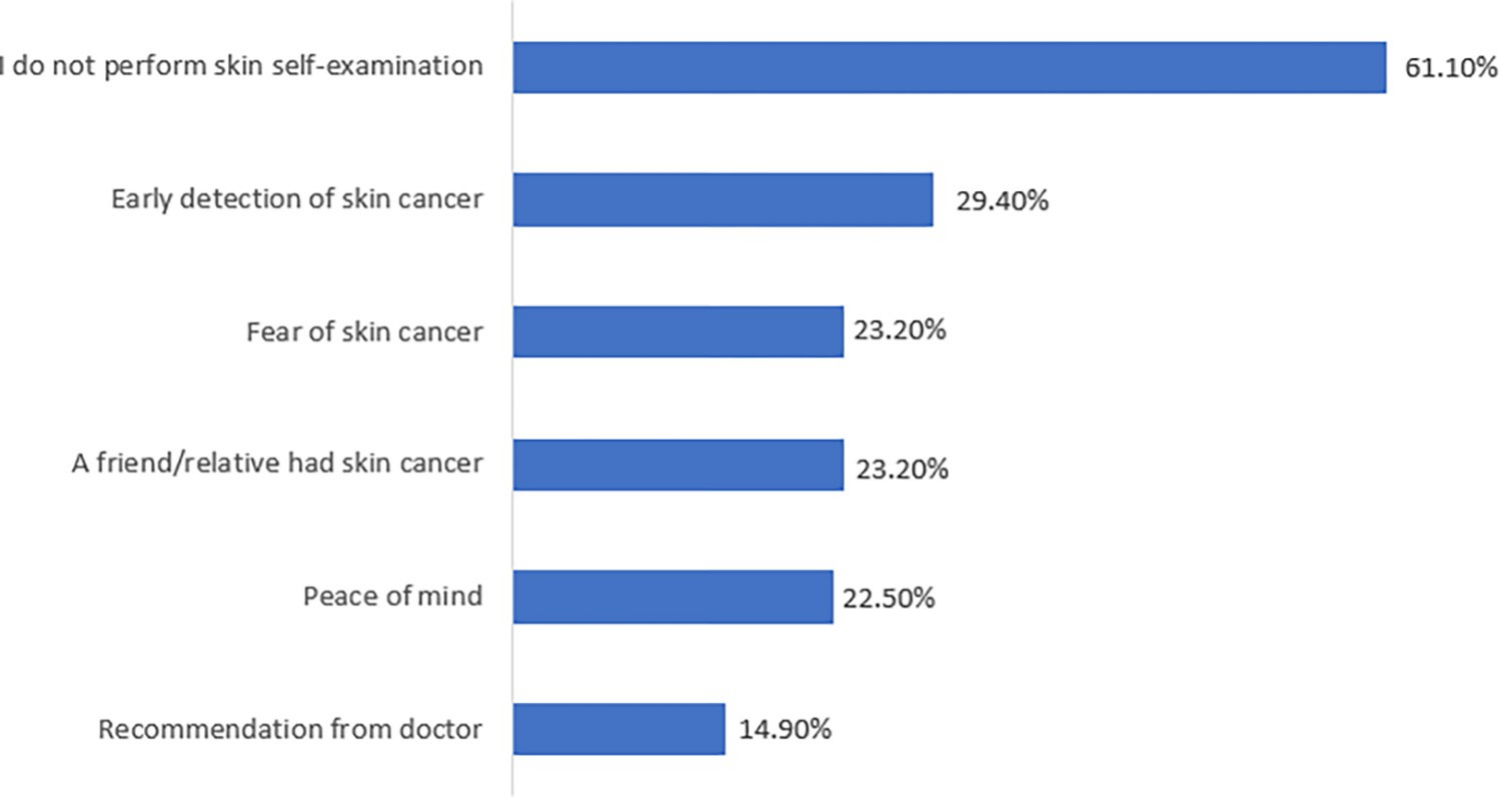
Reasons for performing skin self-examination.

Results revealed that 61.1% of participants reported not performing skin self-examinations. Among those who did perform them, 31.3% cited early detection of skin cancer as their reason; while 15.3% stated that, a doctor recommended it (Fig 2).

Participants’ knowledge about skin cancer and its screening is presented in Table 2. The most correctly answered item was “skin cancer is a fatal disease and can lead to death.” (77.6%), followed by “skin cancer is contagious” (67.1%). On the other hand, the least correctly answered item was “the treatment of skin cancer can only be performed by surgery” (37.9%). The median of the knowledge score was 4 (3–6) out of a maximum possible score of 8.

**Table 2:** Knowledge about skin cancer and skin cancer screening.

	No	I don’t know	Yes
Skin cancer is a fatal disease and can lead to death. *	50 (5.4%)	157 (17%)	717 (77.6%)
Skin cancer is contagious. **	620 (67.1%)	190 (20.6%)	114 (12.3%)
The protection against skin cancer starts in childhood. *	302 (30.2%)	215 (21.5%)	484 (48.4%)
The self-examination of individuals is vital to notice signs of skin cancer. *	286 (31%)	199 (21.5%)	439 (47.5%)
Recovery rates increase when skin cancer is detected in the early stages. *	286 (31%)	199 (21.5%)	439 (47.5%)
Skin cancer occurs only in sun-exposed areas. *	156 (16.9%)	186 (20.1%)	582 (63%)
Sunscreens help protect against skin cancer. *	264 (28.6%)	180 (19.5%)	480 (51.9%)
The treatment of skin cancer can only be performed by surgery. *	253 (27.4%)	321 (34.7%)	350 (37.9%)
Knowledge score [Median (IQR)]	4 (3–6)		

*Yes is the correct answer.

**No is the correct answer. Skin cancer is not contagious because cancer cells from someone with cancer are not able to live in the body of another healthy person.

**Fig 3:**
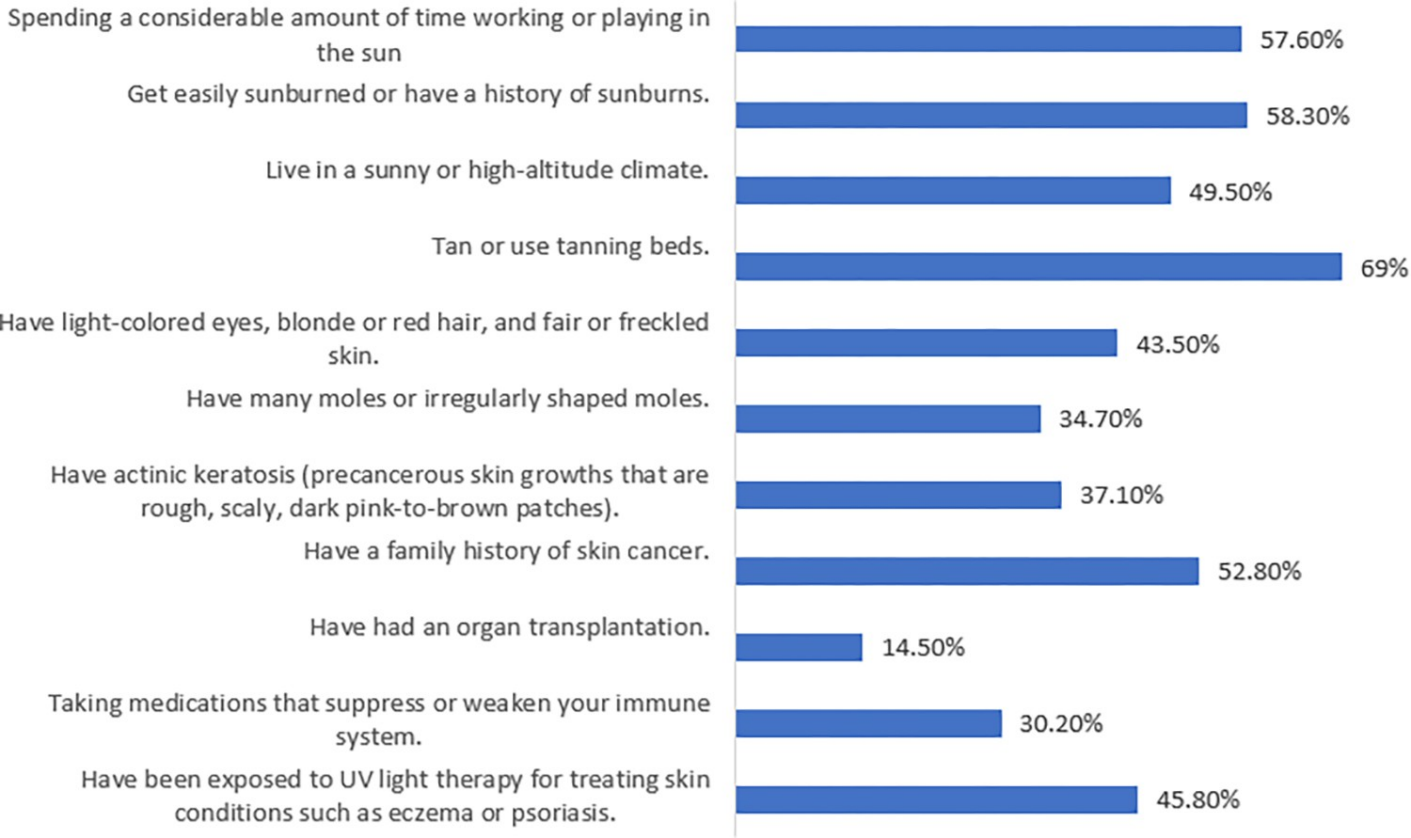
Awareness of risk factors for skin cancer.

Awareness of the risk factors for skin cancer is presented in Fig 3. The most frequently recognized risk factor was “tanning or using tanning beds” (69.1%), and “getting easily sunburned or having a history of sunburns” (58.7%), while the least mentioned risk factor was “having had an organ transplantation” (14.9%).

**Fig 4:**
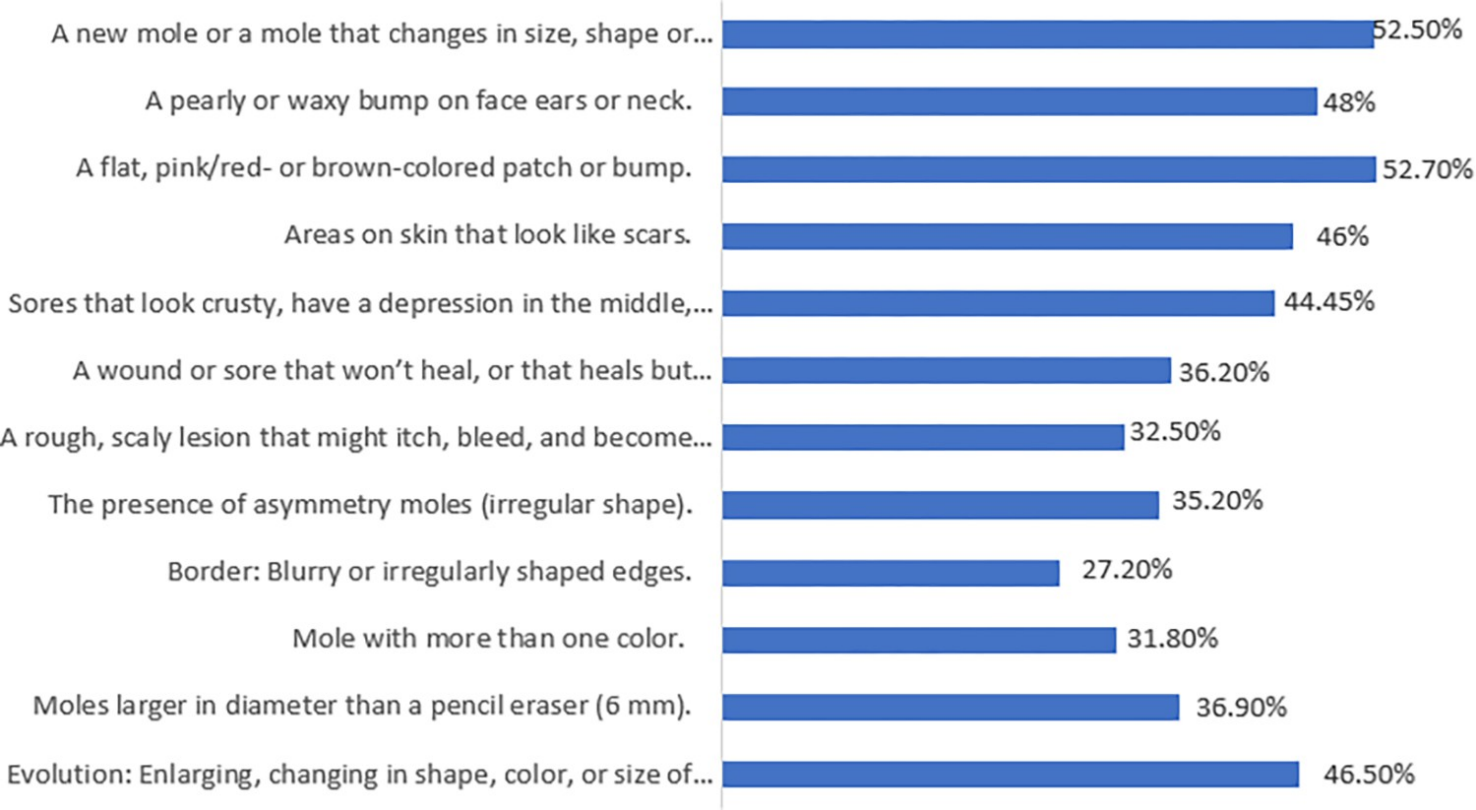
Awareness of signs and symptoms associated with skin cancer.

Awareness of the signs and symptoms associated with skin cancer is displayed in Fig 4. The most commonly recognized signs and symptoms included "a flat, pink/red or brown-colored patch or bump” (53.6%), “a new mole or a mole that changes in size, shape or color, or that bleeds” (51.9%), and”a pearly or waxy bump on the face, ears or neck” (48.8%). The least recognized sign or symptom was “Border: Blurry or irregularly shaped edges” (27.0%).

As shown in Table 3, the participants demonstrated positive attitudes, particularly regarding the perceived importance of knowing about skin cancer (73.3%), and taking precautions against the damaging effects of the sun (67.8%). In addition, 30.7% of participants disagreed that someone who has cancer is simply an unlucky person, and 24.2% did not find it difficult to protect themselves from the sun. The median of the attitude score was 37 (33–39) out of a maximum possible score of 50.

**Table 3:** Attitudes towards skin cancer screening.

	Strongly disagree	Disagree	Neutral	Agree	Strongly agree
It is important for me to know about skin cancer.	74 (8.0%)	16 (1.7%)	156 (16.9%)	490 (53.0%)	188 (20.3%)
Someone who has cancer is just an unlucky person. *	121 (13.1%)	163 (17.6%)	283 (30.6%)	147 (15.9%)	210 (22.7%)
Skin cancer screening should be implemented on a large scale.	66 (7.1%)	51 (5.5%)	216 (23.4%)	377 (40.8%)	214 (23.2%)
I believe that skin cancer screening is effective in preventing skin cancer.	49 (5.3%)	68 (7.4%)	197 (21.3%)	288 (31.2%)	322 (34.8%)
If skin cancer is diagnosed in an early stage, the treatment outcomes can be better.	77 (8.3%)	46 (5.0%)	180 (19.5%)	356 (38.5%)	265 (28.7%)
Regular physical/clinical examination by a dermatologist helps detect the early stages of skin cancer.	51 (5.5%)	76 (8.2%)	175 (18.9%)	300 (32.5%)	322 (34.8%)
Self-checks for skin cancer help to detect the early signs of skin cancer.	57 (6.2%)	46 (5.0%)	200 (21.6%)	368 (39.8%)	253 (27.4%)
Public awareness about skin cancer is important to reduce the risk.	52 (5.6%)	74 (8.0%)	181 (19.6%)	290 (31.4%)	327 (35.4%)
All people should take precautions against the damaging effects of the sun.	63 (6.8%)	49 (5.3%)	186 (20.1%)	351 (38.0%)	275 (29.8%)
I find it difficult to protect myself from the sun*	57 (6.2%)	166 (18.0%)	250 (27.1%)	207(22.4%)	244 (26.4%)
Attitude score [Median (IQR)]	37 (33–39)				

*Reversed coded items.

As shown in Table 4, the most commonly reported practices included wearing long pants and a long-sleeved shirt during the summer (45.9%), applying sunscreen half an hour before going outside (36.6%), and using broad-spectrum sunscreen with an SPF of 30 or higher (35.6%). The least reported practice was consulting a dermatologist if any changes in the skin occur (21.3%). The median of the practice score was 41 (31–45) out of a maximum possible score of70.

**Table 4:** Skin cancer screening practice.

	Never	Rarely	Sometimes	Often	Always
I avoid the sun during the hours of 10 am to 4 pm.	86 (9.3%)	158 (17.1%)	368 (39.8%)	236 (25.5%)	76 (8.2%)
When I go out, I wear hats with wide brims to protect my face and ears.	123 (13.3%)	190 (20.6%)	338 (36.6%)	219 (23.7%)	54 (5.8%)
I wear sunglasses, and glasses that block both UV-B and UV-A rays.	107 (11.6%)	166 (18%)	406 (43.9%)	174 (18.8%)	71 (7.7%)
I use a broad-spectrum sunscreen with a skin protection factor (SPF) of 30 or higher.	130 (14.1%)	242 (26.2%)	223 (24.1%)	217 (23.5%)	112 (12.1%)
I apply the sunscreen 30 minutes before I go outside.	184 (19.9%)	161 (17.4%)	241 (26.1%)	198 (21.4%)	140 (15.2%)
I wear sunscreen every day, even on cloudy days and during winter.	163 (17.6%)	204 (22.1%)	221 (23.9%)	219 (23.7%)	117 (12.7%)
I use a lip balm with sunscreen.	177 (19.2%)	170 (18.4%)	259 (28%)	187 (20.2%)	131 (14.2%)
I wear long pants and a long-sleeved shirt during summer.	119 (12.9%)	144 (15.6%)	237 (25.6%)	244 (26.4%)	180 (19.5%)
I look for clothing with an ultraviolet protection factor label for extra protection.	330 (35.7%)	190 (20.6%)	182 (19.7%)	139 (15%)	83 (9%)
If I want a tanned look, I avoid tanning beds and I use a spray-on tanning product instead.	229 (24.8%)	182 (19.7%)	260 (28.1%)	175 (18.9%)	78 (8.4%)
I check all my skin for any changes in size, shape or color of skin growths or the development of new skin spots.	199 (21.5%)	210 (22.7%)	278 (30.1%)	161 (17.4%)	76 (8.2%)
I use mirrors and even take pictures to help identify changes in my skin over time.	325 (35.2%)	210 (22.7%)	176 (19%)	153 (16.6%)	60 (6.5%)
I make an appointment for a full-body skin exam with the dermatologist if I notice any changes in a mole or other skin spot.	317 (34.3%)	200 (21.6%)	210 (22.7%)	133 (14.4%)	64 (6.9%)
I ask my healthcare provider or pharmacist if any of the medications I take make my skin more sensitive to sunlight.	254 (27.5%)	205 (22.2%)	255 (27.6%)	150 (16.2%)	60 (6.5%)
Practice score [Median (IQR)]	41 (31–45)				

**Fig 5:**
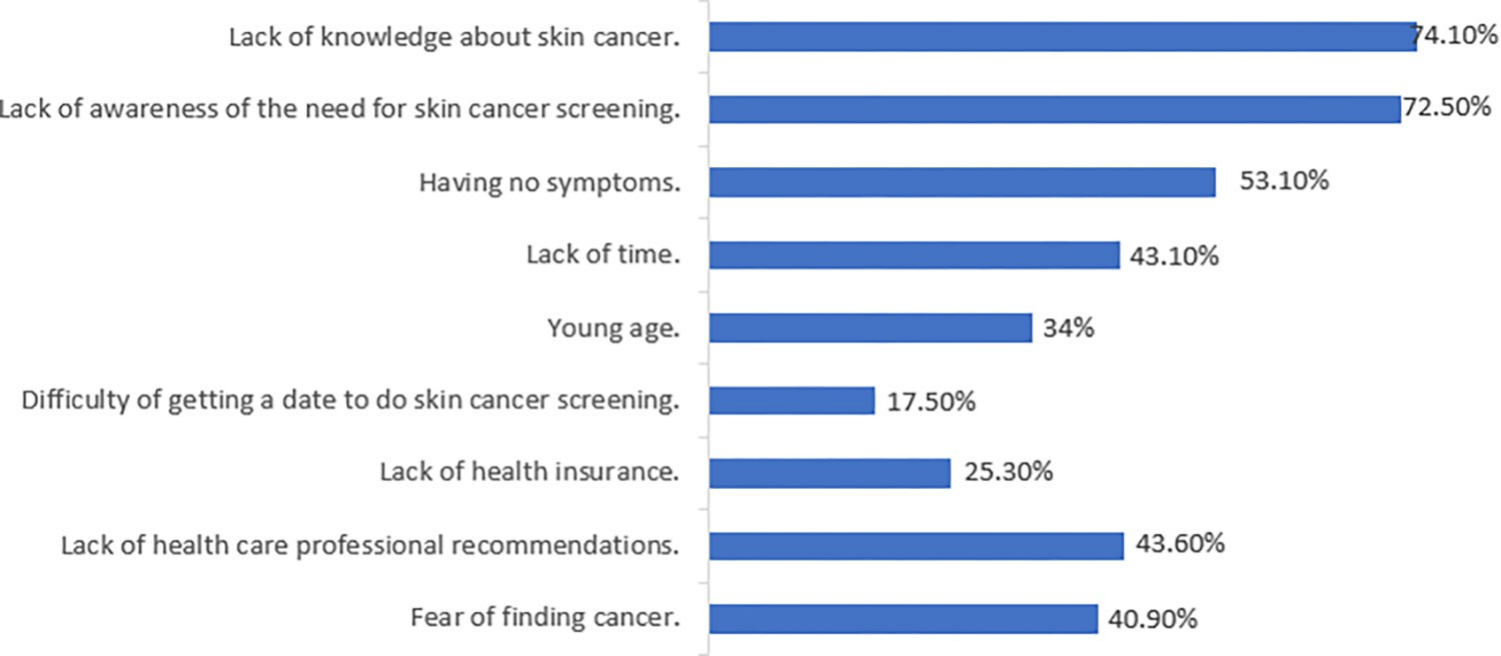
The barriers to skin cancer screening.

As shown in Fig 5, the most frequently reported barriers to skin cancer screening were "lack of knowledge about skin cancer” (74.1%), “lack of awareness of the need for skin cancer screening” (72.2%), and “having no symptoms” (54.1%), while the least frequently reported barrier was “difficulty of getting a date to do skin cancer screening” (16.8%).

As shown in Table 5, the logistic regression revealed that older participants (OR = 0.985, 95%CI: 0.971–0.998, p = 0.025), and participants who did not have health insurance (OR = 0.478, 95%CI: 0.301–0.758, p = 0.002) showed significantly lower odds of being in the high practice group when compared with their counterparts. Female participants (OR = 1.833, 95%CI: 1.361–2.469, p < 0.001) and married individuals (OR = 1.415, 95%CI: 1.006–1.992, p = 0.046) reported higher odds of being in the high practice group than male participants and single individuals, respectively. In addition, individuals who did not smoke (OR = 1.568, 95%CI: 1.045–2.352, p = 0.03) or stopped smoking (OR = 2.555, 95% CI: 1.082–6.034, p = 0.032), reported higher odds of being in the high practice group when compared with smoking individuals. Finally, while participants with higher knowledge levels reported lower odds to screening for skin cancer (OR = 0.908, 95%CI: 0.835–0.989, p = 0.026), those who reported higher attitude scores reported significantly higher odds of being in the high practice group (OR = 1.071, 95%CI: 1.044–1.096, p < 0.001). The Hosmer and Lemeshow goodness-of- fit test indicated a p value of 0.1, confirming the fitness of the model, as there is no significance difference between the observed and model-predicted values.

**Table 5:** Variables associated with skin cancer screening practices (low practice vs. high practice).

		OR	P-value	95% Confidence Interval	
				Lower	Upper
Age		0.985	0.025	0.971	0.998
Gender	Female versus male	1.833	0	1.361	2.469
Marital Status	Married versus single	1.415	0.046	1.006	1.992
Educational Level	High school or less	0.581	0.073	0.32	1.053
	Diploma	0.61	0.11	0.333	1.119
	Bachelor	0.629	0.063	0.387	1.025
	Postgraduates (Master/PhD)	Reference			
Income	No income	1.049	0.85	0.641	1.715
	< 10,000 AED	1.554	0.078	0.951	2.537
	10,000AED to 20,000 AED	1.399	0.15	0.886	2.21
	>20,000 AED	Reference			
Health insurance coverage	No versus Yes	0.478	0.002	0.301	0.758
Smoking	No	1.568	0.03	1.045	2.352
	Quitted Smoking	2.555	0.032	1.082	6.034
	Yes	Reference			
Source of information	Non-medical sources versus medical sources	1.27	0.423	0.708	2.279
Knowledge score		0.908	0.026	0.835	0.989
Attitude score		1.07	<0.001	1.044	1.096

## Discussion

The present study provides insights into knowledge gaps, misconceptions and barriers to skin cancer screening among the population in the UAE. The findings of this study will help identify knowledge gaps and guide the development of public health initiatives to increase awareness and practice of skin cancer screening. Additionally, the study will inform the design of interventions to overcome barriers to the implementation of skin cancer screening.

In line with earlier research findings [26–28], the internet was the most commonly used source for obtaining information on skin cancer in the present study. In addition, half of the participants relied on friends or relatives for such information, which could lead to inaccurate or incorrect information. Social media was the most common information source in a cross-sectional study examining the knowledge of the causes of skin cancer and awareness of preventative measures in Saudi Arabia [15]. This highlights the importance of educating people on the need to obtain information from reliable scientific sources, such as scientific books or reputable websites, to avoid misinformation.

The current study found that the vast majority of participants had never performed skin cancer screening tests (97.6%). Among those who did screen, 29.4% cited early detection of skin cancer as their motivation, while only 14.9% reported that it was recommended by a doctor. Therefore, conducting targeted awareness campaigns to highlight the value of routine skin self-examinations for early detection and encouraging medical professionals to regularly suggest skin self-examinations during patient visits are essential.

Consistent with the results of an earlier study [20], the current study participants exhibited a moderate level of knowledge regarding skin cancer and its screening. In comparison to studies from the Gulf region, one study indicated that while public awareness of skin cancer is relatively high in Saudi Arabia, knowledge of the specific details of the disease remains limited [29]. Another study reported that 53.7% of the parents did not have knowledge about skin cancer or skin self-examination in Turkey [30]. Another study conducted in Saudi Arabia revealed that while most participants were aware of the link between skin cancer and sun exposure, they did not recognize the risks associated with moderate tanning [16]. In contrast, a systematic review observed moderate-to-high levels of skin cancer knowledge among medical students [31]. Proper knowledge about skin cancer and its risk factors was also reported among the medical students in Jordan [32]. This is justifiable, given their extensive medical education and comprehensive background in various types of cancer, including skin cancer. Another study conducted in Turkey revealed that primary care providers had inconsistent levels of knowledge regarding skin cancer and skin self-examination. Specifically, they possessed adequate knowledge of skin cancer symptoms and skin self-examination but lacked sufficient understanding of skin cancer risk factors [33]. The participants in the current study demonstrated knowledge gaps and information needs in several aspects of skin cancer screening, with an obvious window for improvement. In particular, in terms of the fact that protection against skin cancer starts in childhood, recovery rates increase when skin cancer is detected in the early stages, skin cancer occurs only in a sun-exposed areas, sunscreens help protect against skin cancer, and the treatment of skin cancer can only be performed by surgery. In contrast to previous research in Saudi Arabia [15], participants in the current study demonstrated poor awareness regarding the signs, symptoms and risk factors for skin cancer. They specifically lacked awareness of risk factors such as living in sunny or high-altitude climate, having light-coloured eyes, blonde or red hair, and fair or freckled skin, having many moles or irregularly shaped moles, having actinic keratosis, organ transplantation, receiving immunosuppressant medications, and receiving UV light therapy. Additionally, they demonstrated poor awareness of all signs and symptoms of skin cancer. Comprehensive public health campaigns that highlight the value of early detection and preventive measures for skin cancer are paramount. These campaigns should draw attention to important risk factors and raise awareness of the wide range of symptoms and indicators linked to skin cancer. In addition, medical professionals should be adequately equipped with the knowledge required to explain these important points to their patients in an understandable and proactive manner, promoting skin cancer screening.

Generally, the participants in the current study showed unfavourable attitude towards skin cancer screening, with only 32.2% and 24.4% disagreeing with the statement that someone with cancer is just an unlucky person, and that they find it difficult to protect themselves from the sun, respectively. In comparison, a previous study reported low attitude scores toward skin cancer among collegiate athletes [22]. Unfavourable attitudes were associated with poor screening practices across all essential skin cancer screening measures, while improved knowledge was not associated with improved practices in the present study. Similarly, research on nursing students found that, despite having a moderate understanding of skin cancer, they did not take the necessary precautions to protect themselves from sun exposure and other risk factors [34]. Therefore, improving knowledge would be of little value if it was not combined with efforts to enhance perceptions and behaviors regarding skin cancer screening and prevention. It is essential to implement targeted campaigns to dispel common misunderstandings and emphasize the value of taking sun protection measures, such as applying sunscreen and wearing protective clothing.

The most frequently reported barriers to skin cancer screening in the current study were a lack of knowledge about skin cancer, a lack of awareness of the need for screening, and the absence of symptoms. Similarly, a focus group study identified low levels of knowledge and awareness as most common barrier to skin examination [35]. Improving public education and awareness about skin cancer and its preventive measures is essential to overcoming these obstacles. It is recommended that extensive educational campaigns be launched using a variety of media platforms, with a focus on diverse populations. These campaigns should dispel any misconceptions and offer comprehensive information about risks, symptoms, and the importance of early detection of skin cancer. Healthcare professionals should also be trained to promote proactive skin examinations and stress the value of screening even in the absence of symptoms. Given the diverse population of the UAE, including non-Arabic speakers and various socio-economic groups, it is crucial to deliver health information in multiple language, tailoring the content to align with cultural norms of the community, along with implementing targeted outreach strategies to enhance accessibility. These educational programs have the potential to ultimately lower the incidence of skin cancer by raising screening rates and promoting early detection.

In the current study, older participants showed a significantly lower practice of skin cancer screening. Similar results were reported in a previous study conducted among an Ecuadorian population [36]. It is possible that older adults are less aware of, and may not fully comprehend the significance of routine skin cancer screenings. Compared to younger populations, they might be less responsive to health campaigns and educational tools that emphasize the importance of screening. Additionally, lack of health insurance was associated with lower screening practices in the current study. Disparities in cancer screenings exist among U.S adults based on health insurance status and type of insurance [37]. Several research studies have indicated that health insurance status and type are among the most important determinants of cancer screening [38]. People without insurance may encounter financial difficulties that hinder regular checkups and preventative care, such as screenings for skin cancer. Hence, those without insurance may postpone or neglect these crucial screenings, which would result in a lower level of practice than among those with health insurance. However, while health insurance coverage can improve access to preventive screenings, individuals may still incur out-of-pocket expenses such as deductibles, copayments, or coinsurance. These costs, which can vary depending on the insurance plan and the specific screening modality chosen, may discourage some insured individuals from undergoing screenings. If individuals perceive these costs as a significant financial burden, they may be less likely to pursue screening, even when insurance coverage is available. On the other hand, female participants in the present study reported higher levels of skin cancer screening practices than male participants, consistent with findings from earlier research [36,39,40]. A recent study revealed that women reported greater confidence that they could check their skin successfully than men [41]. In some cultures, women prioritize skin care and seek regular screenings to maintain beauty and appearance, influenced by societal norms. Additionally, because skin-related health initiatives and educational campaigns primarily focus on women’s health, this may improve their health literacy and access to information about skin cancer, ultimately leading to improved screening practices. Consistent with the retrospective data analysis of the 2010 Behavioural Risk Factor Surveillance System survey [42], the current study revealed that married individuals reported higher screening practice scores compared to single individuals. This association may be attributed to encouragement from their partners and the increased desire to maintain health for the sake of family and children [43]. Moreover, the present study found that participants who did not smoke or had quit smoking had significantly a higher levels of skin cancer screening practices than those who smoked. This is likely due to their greater concern for their health, which justifies their higher practice levels. Finally, a more positive attitude toward skin cancer screening was associated with higher engagement in screening practices. Positive attitudes likely contribute to increased awareness and perceptions of the procedure’s importance. As a result, individuals with more positive attitudes toward screening are more likely to actively seek out and adhere to recommended screening guidelines. Targeted education campaigns and health promotion initiatives that focus on raising awareness and understanding of skin cancer screening are urgently needed. Interventions specifically designed to address the barriers faced by older adults, males, smokers, and those without health insurance could improve adherence to routine screenings. Additionally, highlighting the health benefits of quitting smoking and promoting positive attitudes toward skin cancer screening can improve screening practices.

It is worth noting that other factors, such as healthcare costs, the stigma associated with misunderstandings about the disease, and living in rural areas with limited access to healthcare facilities, may discourage individuals from seeking skin cancer screening. Additionally, the role of healthcare providers should not be overlooked; equipping them with the necessary knowledge and skills regarding preventive care could help enhance skin cancer screening rates within the community. Future research that explores cultural, psychosocial, socioeconomic, systemic, and healthcare-related factors in depth could provide a better understanding of the obstacles to skin cancer screening and inform targeted interventions to enhance these practices among the public.

This study has several limitations. The cause-and-effect relationship cannot be verified due to the cross-sectional study design. Additionally, the study lacks demographic details such as nationality, ethnicity and geographic distribution within the UAE, which may limit the representativeness of the sample and affect the generalizability of the findings. While we used multiple platforms and tracked response rates to enhance the demographic diversity of the participants and address any imbalances accordingly, the use of convenience sampling and online platforms may still limit the generalizability of the findings, as the participants may not fully reflect the diversity of the entire population. Future research employing more rigorous sampling methods, such as random sampling, could improve the representativeness of the study findings. Furthermore, although the survey in this study collected anonymous responses and did not gather personal identifiers, the use of self-report questionnaires may increase the potential for social desirability bias, where respondents provide answers they believe will make them appear more health-conscious. A more thorough evaluation of this limitation, particularly by comparing self-reported data with objective measures in future studies would be beneficial. Lastly, although the websites references used in this study are reputable and provide valuable facts about skin cancer that are relevant to our study, and offer up-to-date information from credible organizations, incorporating more peer-reviewed journal articles and fewer general web pages would strengthen the research’s academic foundation.

## Conclusions

The current study revealed moderate knowledge, an unfavourable attitude, and inadequate practices regarding skin cancer screening, along with several identified barriers. Therefore, it is recommended to organize virtual sessions and online awareness campaigns via social media platforms, led by expert dermatologists or healthcare providers, to address misconceptions, provide guidance on how to detect skin changes, and clarify the benefits of early detection. These virtual sessions will increase audience accessibility and ensure broader outreach. Additionally, implementing in-person awareness campaigns that include distribution of informative brochures, free skin checks and interactive displays at community canters, malls, and public events could improve awareness, address misconceptions, and enhance screening practices. Notably, there is a need for government policies that incentivize individuals who seek preventive care and strengthen the role of healthcare providers in promoting screening practices to prevent skin cancer. These initiatives should specifically target older adults, men, smokers, and individuals without health insurance. They may also be effective in enhancing skin cancer screening practices among low-income groups, individuals living in rural areas, and those with limited access to healthcare facilities. Future longitudinal studies are needed to assess how public awareness of skin cancer screening evolves following targeted educational interventions.

## Supporting information

S1 Data The survey dataset for participants’ responses. (XLSX)
